# NGSTroubleFinder: a tool for detection and quantification of contamination and kinship across human NGS data

**DOI:** 10.1093/nargab/lqag006

**Published:** 2026-01-27

**Authors:** Samuel Valentini, Tecla Venturelli, Xavier Gallego, Lynn Durham, Laura Perez-Cano, Emre Guney

**Affiliations:** STALICLA Discovery and Data Science Unit, World Trade Center, Moll de Barcelona, Edif Este, 08039 Barcelona, Spain; STALICLA Discovery and Data Science Unit, World Trade Center, Moll de Barcelona, Edif Este, 08039 Barcelona, Spain; STALICLA Discovery and Data Science Unit, World Trade Center, Moll de Barcelona, Edif Este, 08039 Barcelona, Spain; STALICLA Discovery and Data Science Unit, World Trade Center, Moll de Barcelona, Edif Este, 08039 Barcelona, Spain; STALICLA Discovery and Data Science Unit, World Trade Center, Moll de Barcelona, Edif Este, 08039 Barcelona, Spain; STALICLA Discovery and Data Science Unit, World Trade Center, Moll de Barcelona, Edif Este, 08039 Barcelona, Spain

## Abstract

Quality control constitutes a critical component of any next-generation sequencing (NGS) pipeline; however, most existing pipelines emphasize technical quality assessment (e.g. read quality, alignment metrics, duplication rates) while overlooking other equally important dimensions, such as sample identity verification, contamination detection, kinship analysis, and metadata concordance. Detecting issues like cross-sample contamination and sample swaps is essential to control data integrity. Here, we present NGSTroubleFinder, a novel tool to detect cross-sample contamination in human whole-genome and whole-transcriptome sequencing data, sample swaps, and mismatches between the reported and the inferred genetic and transcriptomic sexes. It can be run directly on BAM/CRAM files without requiring additional variant-calling steps and offers an integrated pipeline for ensuring quality control on NGS data, generated particularly within the context of clinical studies or research projects involving family members. It produces a detailed report that combines the results of its multiple analyses, including kinship, sex prediction, and contamination metrics. The tool reports extensive information on the samples, both in textual and HTML formats, including key plots for easy interpretation of the results. NGSTroubleFinder is written in Python and incorporates a custom-built parallelized pileup engine written in C, and it can be easily installed with pip. The tool source code and the models are freely available on GitHub (https://github.com/STALICLA-RnD/NGSTroubleFinder), and a containerized version is available on Docker Hub (https://hub.docker.com/r/staliclarnd/ngstroublefinder).

## Introduction

Next-generation sequencing (NGS) has revolutionized genomics, transcriptomics, and epigenomics by generating vast amounts of data that provide unprecedented insights into biological systems. However, the validity of these insights heavily depends on the integrity of the sequencing data, making quality control (QC) a fundamental step in NGS data analysis. Contamination in NGS samples, whether from exogenous sources or cross-sample contamination, is not uncommon due to the laborious experimental steps prone to human error. Such contamination can significantly skew results and lead to erroneous conclusions. Tools like MultiQC [1] allow for fast and reliable identification of issues related to the technical quality of a sequencing experiment, like read quality, duplication, and alignment statistics. A higher-than-expected ratio of unmapped reads, followed by alignment of some of these reads, is often sufficient to pinpoint potential viral and bacterial contamination [2–4]. However, post-alignment QC to identify issues like sample swaps and cross-sample contamination is often overlooked and not typically included in standard QC procedures [5].

Several tools analyzing allelic ratios, k-mer frequencies, or gene markers, such as VerifyBamID2 [6] and read_haps [7], have been developed to identify and quantify contamination. Other tools, like ContEst, Conpair, Vanquish, ART-DeCo, CleanCall, VICES, MICon, and CHARR, have been benchmarked in [8]. However, several of the aforementioned tools exhibited execution limitations, and none of them demonstrated applicability to RNA-sequencing data analysis.

On the other hand, tools like Somalier [9] and NGSCheckmate [10] are often employed to identify kinship and sample swaps. However, the integration of these tools into QC pipelines is hindered by the need to install multiple tools with different requirements, and the necessity for substantial technical expertise to effectively utilize and interpret the results.

Here, we present NGSTroubleFinder, a command-line application developed to provide standardized QC for NGS data, with particular utility in clinical and family-based research contexts. A key feature of the tool is its capacity to operate directly on BAM/CRAM files, eliminating the need for additional variant-calling procedures.

The software accommodates both Whole-Genome Sequencing (WGS) and Whole-Transcriptome Sequencing (WTS) data and is specifically designed to support mixed WGS/WTS datasets originating from the same individual or from individuals with familial relatedness. This flexibility allows for comprehensive quality assessment across diverse study designs, including those involving multiple family members or heterogeneous data types.

**Figure 1.**
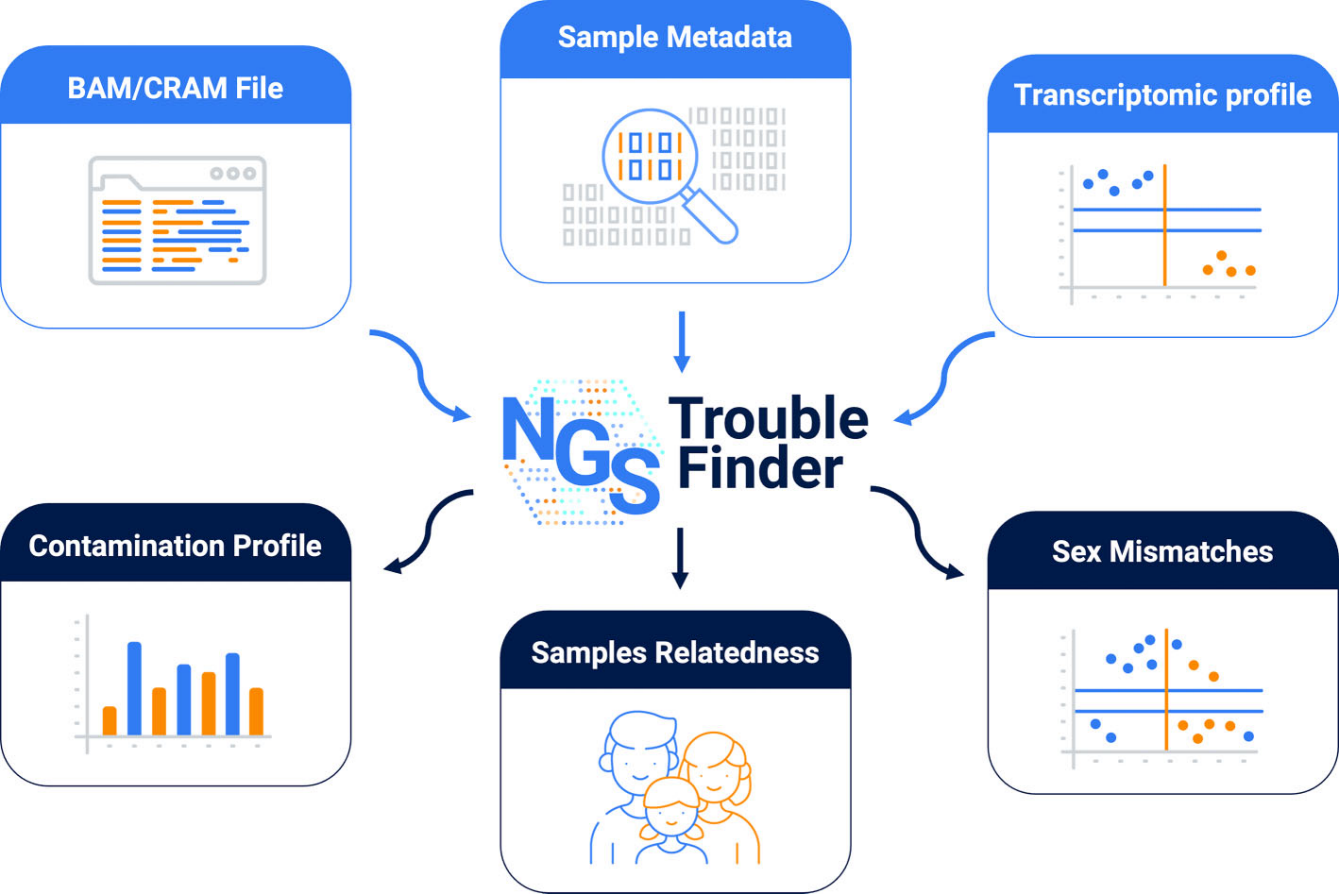
NGSTroubleFinder input and output. NGSTroubleFinder takes as input the BAM/CRAM files for a cohort, the metadata of a cohort, and the transcriptomic profile of the sample (if available). It reports the contamination profile of the samples, their relatedness, and if there are any mismatches on the reported sexes.

NGSTroubleFinder (Fig. 1) integrates smoothly into existing analysis pipelines, including those implemented in workflow languages such as Nextflow, and produces detailed reports that are interactively accessible via standard web browsers. The analytical framework relies on a linear regression model that incorporates information from common variants to quantify the extent of cross-sample contamination and to infer kinship relationships and potential sample swaps. In addition, it evaluates concordance between inferred biological sex, derived from both variant and transcriptomic data, and the sex recorded in accompanying metadata.

By consolidating contamination estimates, kinship analysis, and sex concordance checks into a single resource, NGSTroubleFinder facilitates the identification of mislabeled, contaminated, or otherwise inconsistent samples, while supporting rigorous QC of mixed WGS/WTS datasets.

## Materials and methods

### Existing tools

Several tools have been developed to address specific aspects of QC in NGS data, including contamination estimation, kinship inference, sample swap detection, and sex identification (Table 1). However, most existing tools are designed for a narrow set of tasks, and some even require specific input formats or workflows. For example, ContEst focuses on contamination in tumor–normal pairs, CHARR estimates contamination within germline cohorts from Variant Call Format (VCFs), and NGSCheckmate is specialized for detecting sample swaps. Similarly, Somalier provides robust kinship and sex inference but does not include contamination estimation. For contamination detection, VerifyBamID has emerged as the de facto standard in germline data, offering broad adoption and compatibility with BAM/CRAM and VCF formats, but it cannot be used for other essential QC checks, such as sample swaps. In contrast, NGSTroubleFinder can assess kinship, sample swaps, and sex mismatches in addition to contamination, and it also runs directly on BAM/CRAM files without requiring additional variant-calling steps and avoids additional dependencies.

**Table 1.** Summary of some existing tools to perform QC on NGS datasets

Tool	Capability	Input	Application
NGSTroublefinder	Contamination estimationKinship analysisSample swapsSex identification	BAM/CRAM	Fast QC checking of a batch of mixed sequencing germline (WGS/RNA-seq)
VerifyBamID2	Contamination estimation	BAM/CRAM VCF Samtools pileup	Contamination detection in germline NGS data
Read_haps	Contamination estimation	BAM/CRAM	Contamination detection in germline NGS data
ContEst	Contamination estimation	Custom format	Contamination detection in normal-tumor pairs
CHARR	Contamination estimation	VCF	Contamination estimation in germline NGS cohorts
Somalier	Kinship analysisSample swapsSex identification	BAM/CRAM VCF	Kinship and sex identification in mixed sequencing, germline, and cancer datasets
NGSCheckmate	Sample swaps	BAM VCF FASTQ	Detect sample swaps in NGS batches

### Curated common variants, pileup engine, genotyping, and haplotype detection

NGSTroubleFinder is based on a curated set of very common (minor allelic frequency between 0.1 and 0.9 in the general population) human single nucleotide polymorphisms (SNPs). Biallelic variants have been extracted from dbSNP (build id 151) [11] and filtered by keeping exonic variants, located at least 15 bases from a known INDEL, and that are not located in hypervariable regions containing tandem repeats. The curated variant set includes 164 767 SNPs, including those on the sexual chromosomes that fall outside the pseudoautosomal regions. Those variants are included in the tool in VCF format and are available in the GitHub repository. NGSTroubleFinder analyzes this set of variants and uses their pileup, heuristic genotype, and haplotypes to estimate the contamination level, the genomic sex, and the kinship between samples (Supplementary Methods).

### Training and test datasets

To estimate contamination, we generated two training datasets, one for WGS and one for WTS, which were used to train a linear regression model. Those datasets were constructed from 50 samples (Supplementary Table S1) randomly selected from individuals of European (EUR) ancestry in the 1000 Genomes Project (1000GP) [12]. These samples served as the reference set and were assumed to be free of exogenous DNA. To generate contaminated datasets, each reference sample was paired with a randomly chosen donor sample, and a controlled fraction of donor reads was introduced into the reference reads to simulate contamination. Contamination levels of 0.5%, 1%, 1.5%, 2%, 2.5%, 3%, 4%, 5%, and 10% were applied systematically. For each contamination level, 50 independent contaminated samples were created, resulting in 450 contaminated BAMs in addition to the 50 contamination-free reference BAMs, for a total of 500 samples.

Three independent test datasets were generated: one from the 1000GP and two from internal WGS and WTS samples, respectively. The 1000GP-derived dataset consisted of 25 samples (Supplementary Table S2) selected from five families (five samples per family) representing different ethnic backgrounds (Supplementary Table S3). Contaminated samples were generated by combining reads from two individuals belonging to different families to ensure cross-ethnic contamination. For each predefined contamination level (0.5%, 1%, 1.5%, 2%, 2.5%, 3%, 4%, 5%, and 10%), 15 synthetic contaminated samples were created, resulting in a total of 135 contaminated samples in addition to the 25 contamination-free references.

The internal WGS dataset was generated from 16 independent samples of EUR ancestry sequenced across two batches. For both batches, DNA was extracted from whole blood samples using the Mag-Bind® Blood & Tissue DNA HDQ 96 Kit. WGS libraries were prepared using the KAPA HyperPlus kit. Sequencing was performed on an Illumina NovaSeq platform across two lanes, generating paired-end 150 bp reads at ∼30 × coverage. Alignment was performed using nf-core/sarek version 2.7 against the human reference genome GRCh38. Following the same admixing procedure, 15 synthetic contaminated samples were created for each contamination level, yielding 135 contaminated samples, in addition to the 16 uncontaminated references.

The internal WTS dataset was generated from 24 samples of EUR ancestry obtained from a single sequencing batch. RNA was extracted from whole blood samples using the PAXgene Blood RNA Kit. Sequencing libraries were prepared with the TruSeq Stranded Total RNA protocol, incorporating Ribo-Zero Globin depletion and ERCC spike-in controls (Mix 1 and Mix 2 at a 1:1 ratio). Sequencing was carried out on the Illumina HiSeq 4000 platform, generating paired-end 150 bp reads with a depth of ∼50 million reads per sample. Read alignment and gene quantification were performed using the nf-core/rnaseq pipeline (v3.7) against the human reference genome GRCh38. Following the same admixing procedure, 15 synthetic contaminated samples were derived per contamination level, producing 135 contaminated samples along with 24 uncontaminated references.

Altogether, the three test datasets comprised 405 contaminated samples and 65 contamination-free reference samples, for a total of 470 samples across WGS and WTS data types.

### Linear regression model

For each sample, three features are extracted from pileups of 164 767 curated variants and used to train the linear regression models:

1. *SNPs in tails*: the proportion of SNPs with an allelic fraction in the ranges [0.05–0.15] or [0.85–0.95], normalized to the total number of genotyped SNPs.
2. *Homozygous SNPs*: the proportion of SNPs with an allelic fraction in the ranges [0–0.02] or [0.98–1], normalized to the total number of genotyped SNPs.
3. *Multiple haplotypes*: the proportion of regions supporting more than two haplotypes, normalized to the total number of genotyped positions.

More details about these features are provided in the Supplementary Methods. The model produces a score corresponding to the inferred level of contamination.

The trained models are distributed in the Open Neural Network Exchange format via the project’s GitHub repository, ensuring findability, accessibility, interoperability, and reusability (FAIR principles) in a platform-independent and freely accessible manner.

### Kinship identification

Kinship is identified by using all the available autosomal variants genotyped by the pileup engine. Kinship estimation follows the model introduced in [13], similarly to the tool Somalier [9].

The following metrics are introduced:

- ibs0 – the number of sites where one sample is homozygous reference (AF < 0.02), and another is homozygous alternative (AF > 0.98).
- ibs2 – the number of sites where the samples have the same genotype.
- sharedHets – the number of sites where both samples are heterozygotes (0.2 ≤ AF ≤ 0.8).
- het_i_ is the number of heterozygous sites for sample i.

Given two samples *i,j* their kinship is defined as


\begin{eqnarray*}
{\mathrm{kinship}}_{i,j} = \frac{{\mathrm{sharedHets} - 2 \cdot ibs0}}{{\min \left( {he{t_i},he{t_j}} \right)}}.
\end{eqnarray*}

Two samples are considered replicates (or twins) if their kinship index is >0.95. Two samples are considered related if the kinship goes from 0.15 to 0.95. Finally, they are considered unrelated for values <0.15.

### Sex mismatch identification

Sample sex was determined using two complementary approaches: a variant-based method and a transcriptome-based method. In the variant-based approach, exonic variants from the curated SNP set located on the X and Y chromosomes are analyzed. Two metrics are computed: the heterozygosity ratio on the X chromosome [14] and the total coverage of Y chromosome variants. Variants are considered if they have a coverage of at least 10 and a base quality of 30. A variant genotype is defined as in the kinship analysis. A sample is classified as female if the X chromosome heterozygosity ratio exceeds 1 and Y coverage is below 10. Conversely, a sample is classified as male if the heterozygosity ratio was <1 and Y coverage was >10. Samples falling outside these thresholds were flagged as anomalies.

The transcriptome-based approach relied on previously established sex-specific biomarkers [15]. Expression counts for four Y-linked genes (*RPS4Y1, EIF1AY, DDX3Y, KDM5D*) and the X-linked gene *XIST* (hereafter referred to as discriminative genes) are quantified. Reference distributions of expression levels were constructed using whole blood RNA-seq data from GTEx (V8) [16], separately for males and females. For each sample, expression values of the Y-linked discriminative genes are summed, and *XIST* expression is evaluated against the reference distributions. A sample was classified as female if *XIST* expression exceeded the first percentile of the female distribution, while summed Y-linked expression was below the 99th percentile of the female distribution. Conversely, a sample is classified as male if Y-linked expression exceeded the first percentile of the male distribution, and *XIST* expression is below the 99th percentile of the male distribution. Samples outside these thresholds are flagged as anomalies, and samples lacking discriminative gene expression data are classified as unknown.

## Results

### Quality control report

NGSTroubleFinder produces extensive reports on the inferred sex (Supplementary Fig. S1), kinship (Supplementary Fig. S2), and pileups (Supplementary Fig. S3). Also, per sample pileups are provided using the format used by PaCBAM [17]. Additionally, the tool reports all the regions where haplotypes can be inferred, flagging the ones where more than two haplotypes are observed. Finally, a complete HTML interactive report is generated that can be used to explore the results. In this report, the samples for which the inferred contamination is higher than 0.01 are marked as contaminated (QC status “CONTAMINATION”). The report also marks samples with a mismatch between the original and the inferred sexes and provides the number of samples that belong to the same family as well as the number of replicates.

### Sex identification

We benchmarked the sex identification algorithms using samples from the 1000 Genomes Project (1000GP). Both variant-based and transcriptomic-based algorithms achieved 100% accuracy when applied to WGS datasets and WTS datasets, respectively. In contrast, the application of the variant-based algorithm to WTS data resulted in a reduced precision of 78%, driven primarily by the misclassification of a subset of female samples (Supplementary Table S1). These results demonstrate that the two algorithms capture complementary information and, when applied in combination, can enhance the robustness of sex determination across sequencing modalities.

### Sample swaps and kinship analysis

To evaluate sample swaps, each DNA sample was matched with its corresponding RNA sample. All DNA–RNA pairs exhibited kinship coefficients ≥ 0.98, consistent with replicate samples from the same individual, and no mismatches were detected (Supplementary Table S1). For kinship inference, we used the known pedigrees provided in the 1000GP test dataset as a benchmark. All 33 expected relationships were correctly identified, including sibling pairs, parent–child trios, grandparent–grandchild links, aunt/uncle–nephew/niece pairs, and one cousin pair. No additional or unexpected relationships were observed (Supplementary Table S2).

### Contamination benchmarking

**Figure 2.**
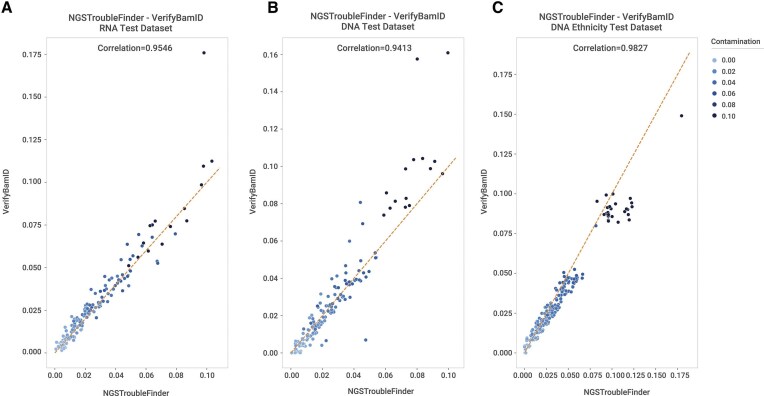
NGSTroubleFinder contamination estimation against VerifyBamID2 estimation. (**A**) Correlation in the RNA test dataset. (**B**) Correlation in the DNA test dataset. (**C**) Correlation in the mixed-ethnicity dataset.

Among pre-existing tools (Table 1), VerifyBamID2 was selected as the comparative benchmark given that it is the de-facto standard for contamination detection. Both tools produce quantitative estimates of contamination, allowing comparison against the benchmark via the mean absolute error metric (Table 2). Overall, NGSTroubleFinder and VerifyBamID2 yielded highly comparable results, with correlations exceeding 94% across all test datasets, indicating a robust generalization of our model (Fig. 2).

**Table 2.** Mean absolute error of estimated contamination using NGSTroubleFinder and VerifyBamID2

Dataset	NGSTroubleFinder train dataset (abs error)	NGSTroubleFinder test dataset (abs error)	VerifyBamID2 test dataset (abs error)
RNA-seq	0.0084	0.0079	0.0083
WGS	0.0033	0.0066	0.0070
Ethnicity	NA	0.0057	0.0035

**Figure 3.**
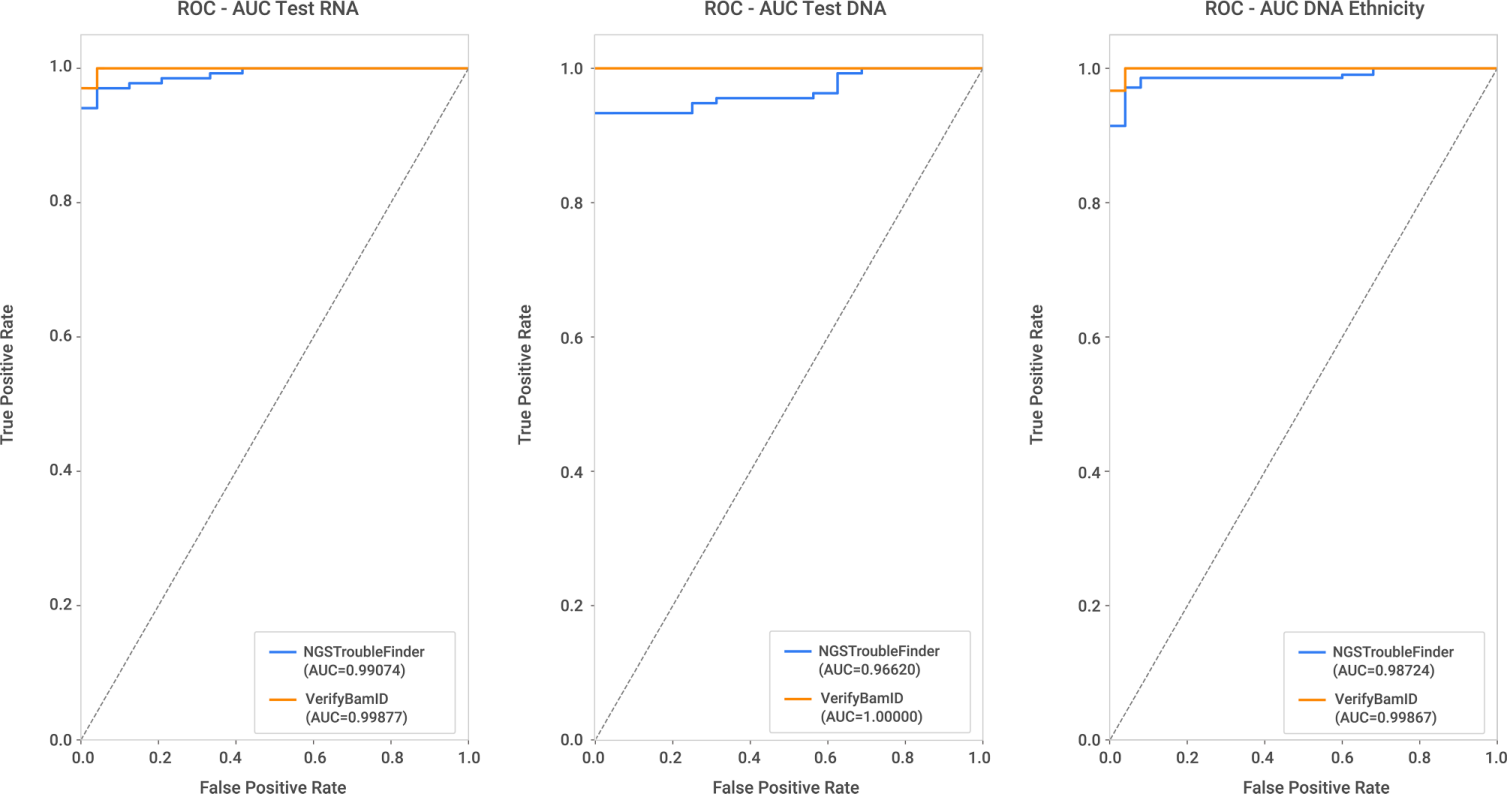
NGSTroubleFinder AUC compared with VerifyBAMID2 AUC in different datasets. (**A**) AUC in the RNA test dataset. (**B**) AUC in the DNA test dataset. (**C**) AUC in the mixed ethnicity dataset.

Analysis of absolute error across contamination levels showed that, for the RNA-seq test dataset, NGSTroubleFinder and VerifyBamID2 perform comparably (Supplementary Table S4). In both tools, the average error increased with higher contamination levels, suggesting that high contamination fractions are inherently more challenging to estimate accurately due to an increase in variance in the allelic frequency of SNPs. This pattern aligns with observations from CHARR [18] and likely reflects increased variance in variant frequencies under higher contamination levels. For DNA datasets, trends were similar, although VerifyBamID2 exhibited slightly lower errors at low contamination levels (Supplementary Table S5) and a higher area under the ROC curve (AUC, Fig. 3). When including samples from multiple ethnicities, NGSTroubleFinder maintained consistent performance, indicating a good generalization of the model, whereas VerifyBamID2 demonstrated slight improvement (Supplementary Table S6). It can also flag relatively low levels of contamination (i.e. ∼1%) in both RNAseq and DNAseq data (Tables S7, S8, S9). Overall, NGSTroubleFinder demonstrates strong contamination estimation capability, with estimates (Fig. 2) and classification performance comparable to VerifyBamID2 even when the data comes from individuals with mixed ethnicity as measured by AUC (Fig. 3).

## Discussion

We present NGSTroubleFinder, a novel easy to use command line tool written in Python for NGS QC. NGSTroubleFinder detects possible sample contamination in WGS and WTS, identifies possible sample swaps when at least two replicates of the same sample are available, and infers kinship. It also determines sample sex using both genomic and transcriptomic methods. Both methods can identify common sex chromosome abnormalities, such as X0 (Turner syndrome) and XXY (Klinefelter syndrome). In Turner syndrome, there is no detectable Y chromosome coverage and no X chromosome heterozygosity; likewise, X- and Y-specific transcripts are absent in the transcriptomic data. In contrast, Klinefelter syndrome is characterized by the presence of Y chromosome coverage, detectable X chromosome heterozygosity, and expression of both X- and Y-discriminative genes. These abnormalities are automatically flagged as anomalies by the tool, although the specific type of abnormality is not differentiated. Other chromosomal abnormalities, such as XXX, XXXX, or XYY, are more challenging to identify, since they require more advanced coverage depth analysis, and their detection is not currently implemented in the tool.

The models currently implemented in NGSTroubleFinder carry certain limitations, as they were trained exclusively on germline samples of EUR genetic background. The tool shows comparable performance in within-ancestry and cross-ancestry comparisons, indicating a good generalization of the model; however, its performance on individuals of mixed ancestry has not yet been assessed and represents a direction of future work. Performance may be limited in cancer samples due to the presence of somatic mutations and copy number alterations. Similarly, its performance may be affected on WES data because of differences in coverage depth. However, custom models tailored for cancer or WES data can be developed and benchmarked. The model is also not suitable to detect inter-species contamination since it relies on human variants. NGSTroubleFinder addresses a critical need for assessing the reliability of NGS data from human samples. The command-line tool integrates seamlessly into NGS data analysis workflows and provides essential information on cross-sample contamination, swaps, familial relationships, and sex mismatches between samples. It generates a user-friendly report that can be visualized in a web browser in a platform-independent manner, and it includes a custom pileup engine to enable efficient computation.

Overall, NGSTroubleFinder demonstrates performance comparable to state-of-the-art tools in identifying sample contamination, while also providing extensive QC information, including the level of estimated contamination, identified sample swaps, family relationships, and sex predicted based on NGS data; furthermore, the tool is user-friendly and available on GitHub and containerized on Docker Hub.

## Supplementary Material

lqag006_Supplemental_File

## Data Availability

The dataset used to train the linear regression, the source code to train the model, and the NGSTroubleFinder source code are all available at https://doi.org/10.5281/zenodo.18170215.
