# Supplementary material for: NGSTroubleFinder: a tool for detection and quantification of contamination and kinship across human NGS data
Source: NAR Genom Bioinform. 2026 Jan 27;8(1):lqag006. doi: 10.1093/nargab/lqag006 (PMC12838523; doi:10.1093/nargab/lqag006)
Supplement: lqag006_Supplemental_File [file lqag006_supplemental_file.pdf]

Supplementary information for NGSTroubleFinder: A tool for detection and quantification of contamination and kinship across human NGS data

Supplementary Methods

NGSTroubleFinder leverages a custom pileup engine written in C and based on the htslib (Bonfield et al., 2021) to compute a pileup of the curated set of variants. The pileup uses a strict quality approach considering a read only if the base quality is at least 30 and its mapping quality is greater than 1. Variants are genotyped using a heuristic approach based on the allelic fraction (AF), where REF and ALT correspond to reference and alternative alleles, respectively.

$$AF = \frac{\#reads \text{ sup}p\, orting\ ALT}{\#reads \text{ sup}p\, orting\ REF + \#\, reads \text{ sup}p\, orting\ ALT}$$

If the AF is less than 0.02 or greater than 0.98 the variant is genotyped as reference homozygous or alternative homozygous respectively. If the variant has an AF from 0.2 to 0.8 it is considered heterozygous. If the variant's AF is outside those ranges, it is considered affected by noise and not called. The variant genotypes are only utilized in kinship detection and not in contamination detection. The tool considers only variants with a coverage of at least 20 in all subsequent computations.

Secondly, close (i.e., less than 150 bases afar) high-quality pairs of variants are used to detect anomalies in the haplotypes following a methodology like read_haps (7). In detail, if a read spans two known variants, only two of the four possible read combinations in a diploid individual (Reference-Reference, Reference-Alternative, Alternative-Reference, and Alternative-Alternative) should be observed. The region is flagged as an anomaly if three or more combinations are observed. A combination is considered observed if at least three non-duplicated reads are supporting the combination.

Supplementary Figures

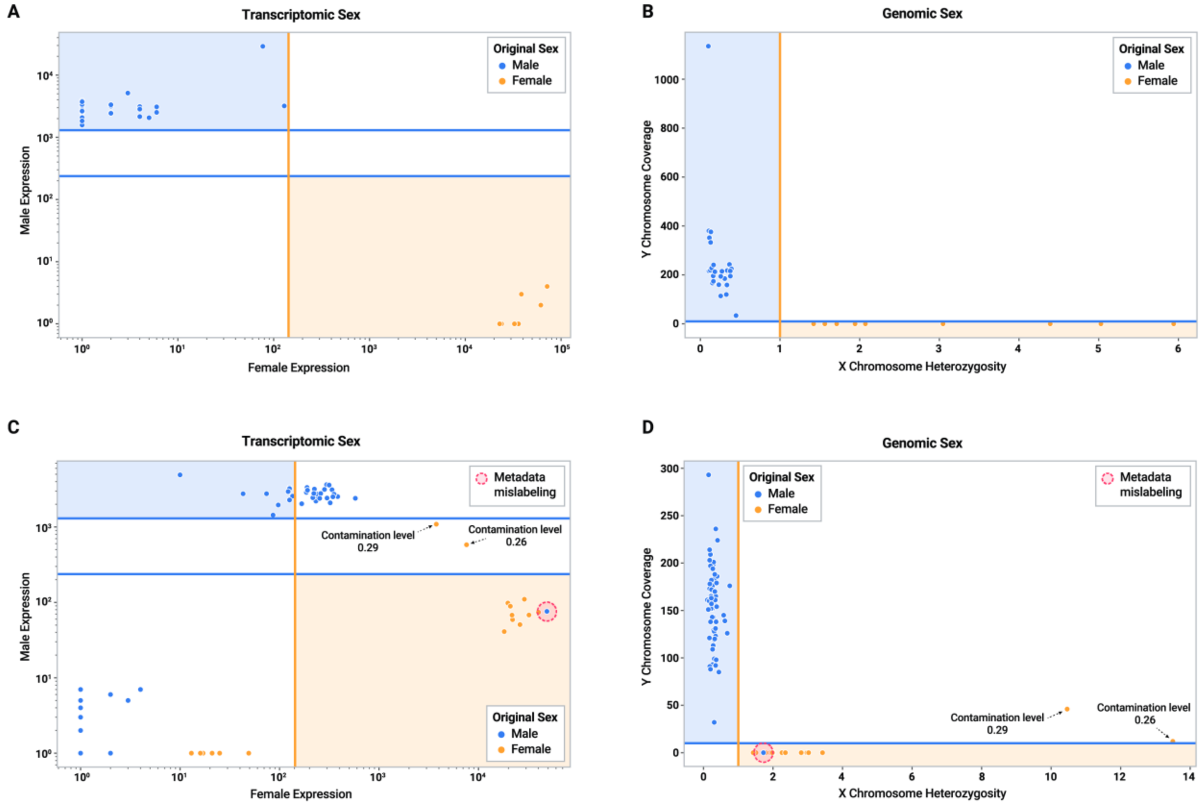

Figure S1: Scatter plots generated using the tool for (A) transcriptomic sex identification and (B) genomic sex identification on the same dataset (38 non-contaminated samples, 7 WGS and 31 WTS). Samples are colored by the user provided sex while the lines and colored areas are providing a guideline on how the samples are classified. The blue region indicates where male samples are expected, the orange region shows where female samples are expected, and the white areas indicate anomalies. The tool achieves perfect classification in both cases. (C) The scatter plot for transcriptomic identification generated using a WTS dataset of 82 contaminated samples (Average Contamination: 0.047, standard deviation: 0.041). Some samples are moved outside the classification thresholds and are marked as anomalies. While only one sample is misannotated in the metadata (circled blue dot), most misclassifications are driven by the cross-sample contamination from male and female samples resulting in a low sex classification precision and recall = 0.23. (D) The scatter plot for genomic sex identification generated using the same contaminated dataset. While the two rightmost samples are highly contaminated and are clear anomalies, the contamination effect is less visible on the other samples. Classification precision = Classification recall = 0.96. The same misannotated sample in the metadata is still present (circled blue dot in the female cluster). While the misannotated sample metadata can be corrected with the usage of the tool, this scenario shows that combining different orthogonal metrics can highlight low quality data in a dataset.

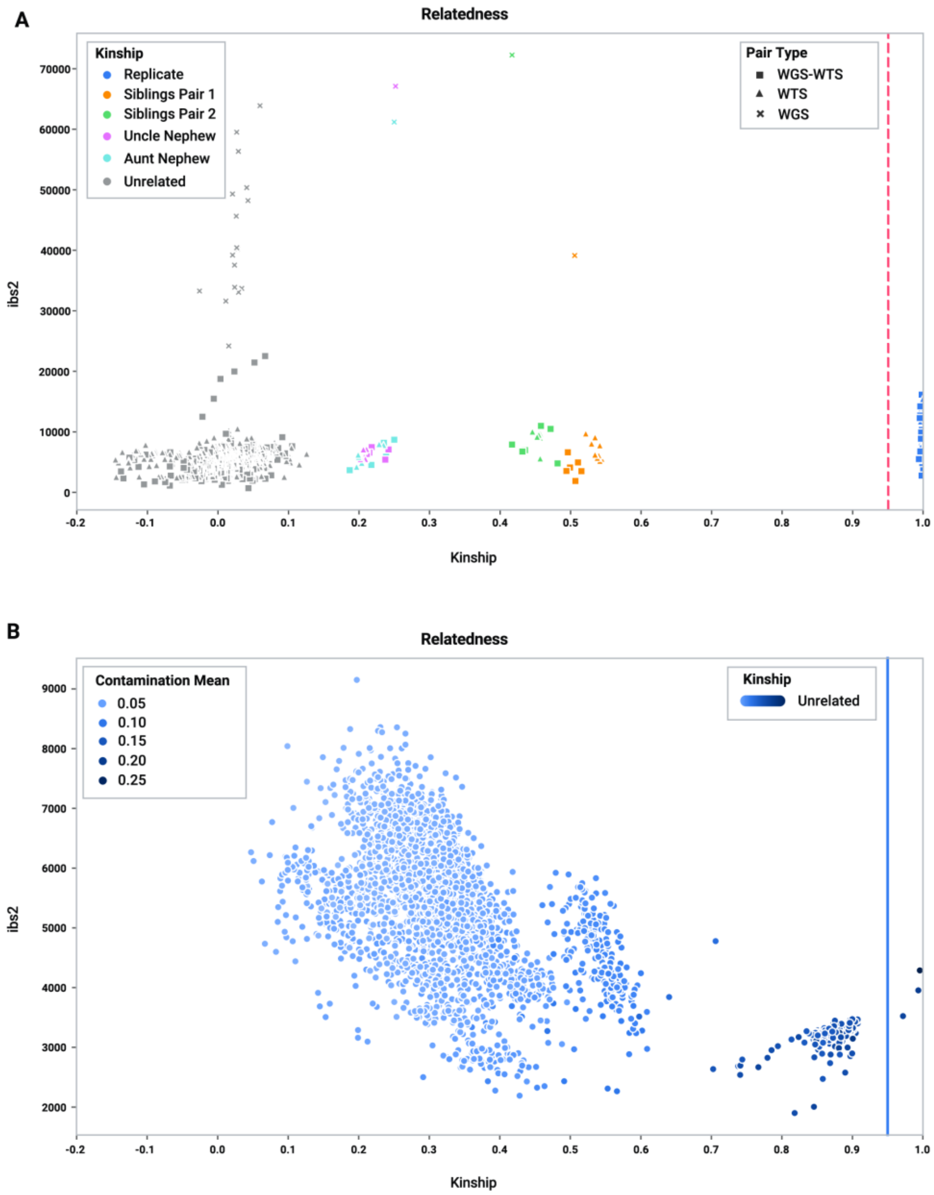

Figure S2: (A) Relatedness plot of a non-contaminated dataset with family relationships (38 samples, 7 WGS and 31 WTS). Each point represents a pair of samples (703 pairs). Pairs are colored by their reported kinship relationship and are marked differently if they belong to WGS, WTS or WGS-WTS pairs. Pairs with high ibs2 (loci where the samples share the same genotype) are WGS while the clusters with low ibs2 are WTS or WGS-WTS pairs since less variants are available to compute the score. The tool is able to infer correctly all the kinship relationships in the dataset. (B) is the same plot as (A) but generated using a highly contaminated dataset (82 WTS samples, 3321 pairs, average Contamination: 0.047, standard deviation: 0.041) without any relationship between the samples. The unrelated cluster is centered in 0.30 because the contamination reduces the high-quality variant available for the computation of the kinship value. The highest contaminated samples are also wrongly identified as replicates/twins. This scenario shows the importance of combining different QC

metrics to interpret the results in an NGS dataset since low quality data can skew different unrelated metrics. The final interpretation of the results must consider all the quality metrics together.

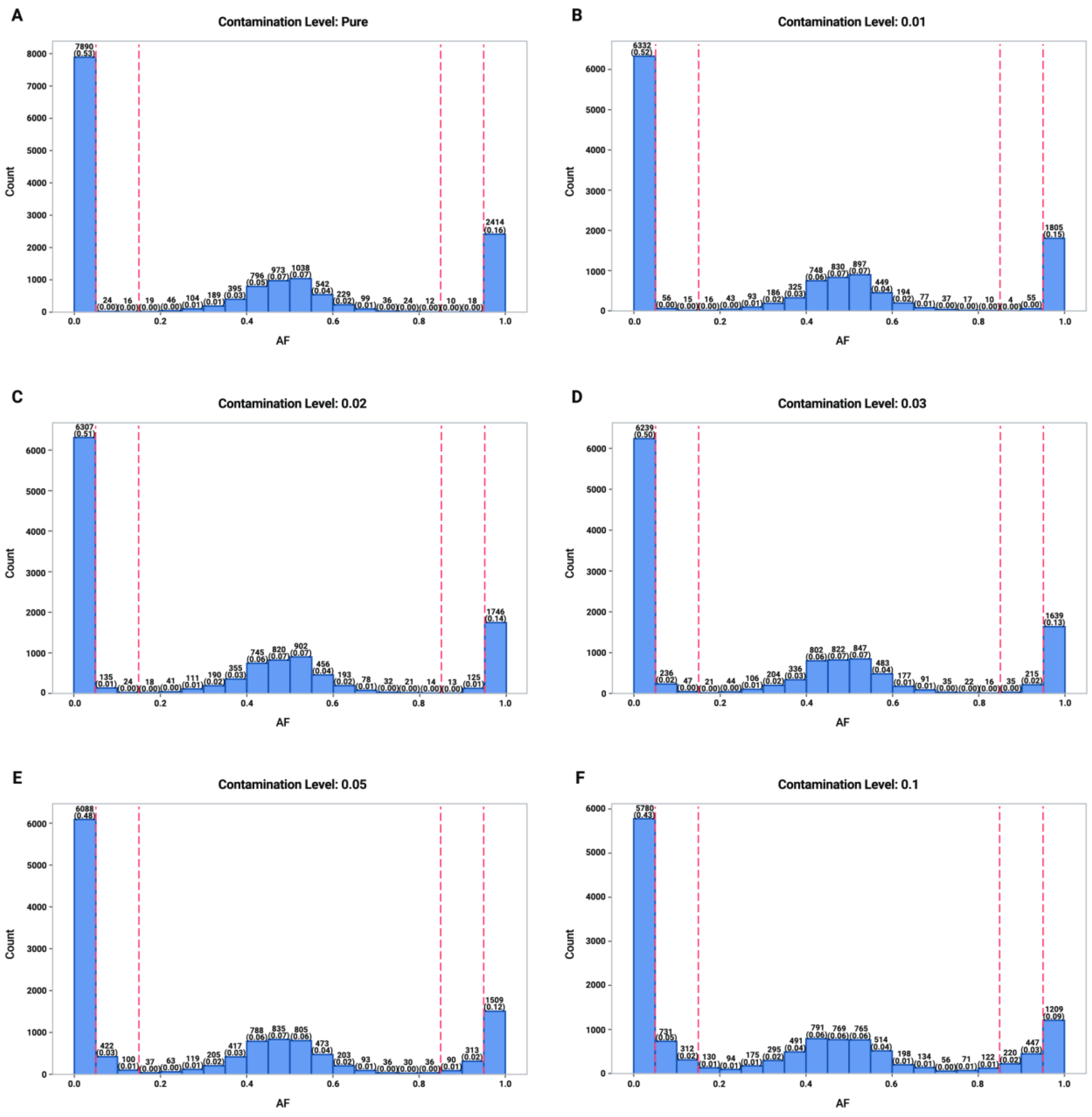

Figure S3: Allelic fraction of the variants with an increasing level of contamination between two WTS samples. (A) pure, (B) 0.01 admixture, (C) 0.02 admixture, (D) 0.03 admixture, (E) 0.05 admixture and (F) 0.1 admixture. Overall, it's possible to see how the number of variants in the [0.05-0.15] and [0.85-0.95] ranges increase as the contamination increases.

Supplementary Tables

Table S1. Information on the 1000GP samples used to build the model. DNA genomic sex is the sex inferred by NGSTroubleFinder using the genomic approach using the WGS of the sample. RNA Genomic sex is the sex inferred using the genomic approach on the WTS of the sample. RNA transcriptomic sex is the sex inferred using the transcriptomic approach on the WTS.

DNA Samples	RNA Samples	Original Sex	DNA Genomic Sex	RNA Genomic Sex	RNA Transcriptomic sex	DNA-RNA kinship
HG00109	HG00109	Male	Male	Male	Male	0.98
HG00127	HG00127	Female	Female	Female	Female	0.99
HG00130	HG00130	Female	Female	Indeterminate	Female	0.99
HG00133	HG00133	Female	Female	Female	Female	0.98
HG00139	HG00139	Male	Male	Male	Male	0.99
HG00146	HG00146	Female	Female	Indeterminate	Female	0.99
HG00154	HG00154	Female	Female	Indeterminate	Female	0.99
HG00233	HG00233	Female	Female	Female	Female	0.99
HG00235	HG00235	Female	Female	Indeterminate	Female	0.98
HG00243	HG00243	Male	Male	Male	Male	0.99
HG00251	HG00251	Male	Male	Male	Male	0.99
HG00253	HG00253	Female	Female	Female	Female	0.98
HG00257	HG00257	Female	Female	Female	Female	0.99
HG00259	HG00259	Female	Female	Female	Female	0.99
HG00262	HG00262	Female	Female	Indeterminate	Female	0.99

HG01334	HG01334	Male	Male	Male	Male	0.99
NA06985	NA06985	Female	Female	Indeterminate	Female	0.98
NA06994	NA06994	Male	Male	Male	Male	0.99
NA07048	NA07048	Male	Male	Male	Male	0.99
NA11843	NA11843	Male	Male	Male	Male	0.99
NA11881	NA11881	Male	Male	Male	Male	0.99
NA11930	NA11930	Male	Male	Male	Male	0.99
NA11995	NA11995	Female	Female	Female	Female	0.99
NA12058	NA12058	Female	Female	Indeterminate	Female	0.99
NA12272	NA12272	Male	Male	Male	Male	0.98
NA12273	NA12273	Female	Female	Indeterminate	Female	0.99
NA12282	NA12282	Male	Male	Male	Male	0.99
NA12342	NA12342	Male	Male	Male	Male	0.99
NA12347	NA12347	Male	Male	Male	Male	0.99
NA12399	NA12399	Male	Male	Male	Male	0.99
NA12750	NA12750	Male	Male	Male	Male	0.99
NA12763	NA12763	Female	Female	Indeterminate	Female	0.98
NA12775	NA12775	Male	Male	Male	Male	0.99
NA12778	NA12778	Female	Female	Female	Female	0.98
NA12814	NA12814	Male	Male	Male	Male	0.99
NA12873	NA12873	Female	Female	Indeterminate	Female	0.99
NA12889	NA12889	Male	Male	Male	Male	0.98

NA20504	NA20504	Female	Female	Indeterminate	Female	0.99
NA20509	NA20509	Male	Male	Male	Male	0.99
NA20512	NA20512	Male	Male	Male	Male	0.99
NA20531	NA20531	Female	Female	Female	Female	0.99
NA20589	NA20589	Female	Female	Female	Female	0.99
NA20752	NA20752	Male	Male	Male	Male	0.99
NA20765	NA20765	Male	Male	Male	Male	0.99
NA20774	NA20774	Female	Female	Female	Female	0.98
NA20787	NA20787	Male	Male	Male	Male	0.99
NA20790	NA20790	Female	Female	Female	Female	0.99
NA20804	NA20804	Female	Female	Female	Female	0.98
NA20826	NA20826	Female	Female	Female	Female	0.99
NA20810	---	Male	Male	---	---	---
---	NA20816	Male	---	Male	Male	---

Table S2. Information on the 1000GP samples used to test the model. Inferred genomic sex is the sex inferred by NGSTroubleFinder using the genomic approach using the WGS of the sample.

DNA Sample	Sex	Inferred Genomic Sex	Ethnicity
HG00656	Male	Male	EAS
HG00657	Female	Female	EAS
HG00658	Male	Male	EAS
HG00702	Female	Female	EAS
HG00703	Male	Male	EAS
HG03943	Male	Male	SAS
HG03944	Female	Female	SAS
HG03950	Male	Male	SAS
HG03992	Male	Male	SAS
HG04036	Female	Female	SAS
NA06986	Male	Male	EUR
NA06997	Female	Female	EUR
NA07045	Female	Female	EUR
NA12801	Male	Male	EUR
NA12813	Female	Female	EUR
NA18861	Female	Female	AFR
NA18862	Male	Male	AFR
NA18863	Male	Male	AFR
NA19660	Female	Female	AMR
NA19661	Male	Male	AMR
NA19662	Female	Female	AMR
NA19685	Male	Male	AMR
NA19686	Female	Female	AMR
NA19713	Female	Female	AFR

NA19983	Female	Female	AFR

Table S3. Relatedness information on the 1000GP samples used to test the kinship inference

Sample1	Sample2	Kinship	True Relationship	Sample 1 inferred sex	Sample 2 inferred sex
NA19662	NA19685	0.55	Siblings	Female	Male
HG03943	HG04036	0.53	Father-Daughter	Male	Female
HG00658	HG00702	0.53	Siblings	Male	Female
HG03944	HG04036	0.52	Mother-Daughter	Female	Female
HG03992	HG04036	0.51	Siblings	Male	Female
NA19661	NA19685	0.51	Father-Son	Male	Male
NA19661	NA19662	0.51	Father-Daughter	Male	Female
HG00702	HG00703	0.51	Mother-Son	Female	Male
NA06986	NA06997	0.50	Father-Daughter	Male	Female
NA19660	NA19662	0.50	Mother-Daughter	Female	Female
NA18862	NA18863	0.50	Father-Son	Male	Male
HG00657	HG00702	0.50	Mother-Daughter	Female	Female
NA18861	NA18863	0.50	Mother-Son	Female	Male
NA19713	NA19983	0.50	Mother-Daughter	Female	Female
NA12801	NA12813	0.50	Son-Mother	Male	Female
HG03944	HG03992	0.50	Mother-Son	Female	Male
HG03943	HG03992	0.50	Father-Son	Male	Male
NA19660	NA19685	0.50	Mother-Son	Female	Male
NA19685	NA19686	0.49	Father-Daughter	Male	Female
NA06997	NA07045	0.49	Daughter-Mother	Female	Female

HG00656	HG00658	0.49	Father-Son	Male	Male
HG00657	HG00658	0.49	Mother-Son	Female	Male
HG00656	HG00702	0.49	Siblings	Male	Female
NA07045	NA12813	0.49	Siblings	Female	Female
NA19661	NA19686	0.32	Grandfather-Granddaughter	Male	Female
HG00658	HG00703	0.31	Uncle-Nephew	Male	Male
NA19662	NA19686	0.28	Aunt-Niece	Female	Female
HG00657	HG00703	0.28	Grandmother-Grandson	Female	Male
NA07045	NA12801	0.26	Aunt-Nephew	Female	Male
NA06997	NA12813	0.25	Nephew-Aunt	Female	Female
HG00656	HG00703	0.22	Grandfather-Grandson	Male	Male
NA19660	NA19686	0.20	Grandmother-Granddaughter	Female	Female
NA06997	NA12801	0.15	Cousins	Female	Male

Table S4. Contamination prediction error (Mean Absolute Error) on the RNA test dataset

Contamination	NgsTroubleFinder Mean Absolute Error (Test)	VerifyBamID2 Mean Absolute Error (Test)
0	0.00477	0.00433
0.005	0.00447	0.00518
0.01	0.00294	0.00363
0.015	0.00536	0.00540
0.02	0.00272	0.00366
0.025	0.00910	0.00856
0.03	0.00785	0.00563
0.04	0.00854	0.00816
0.05	0.01037	0.01178
0.1	0.02508	0.02940

Table S5. Contamination prediction error (Mean Absolute Error) on the DNA test dataset

Contamination	NgsTroubleFinder Mean Absolute Error (Test)	VerifyBamID2 Mean Absolute Error (Test)
0	0.00424	0.00049
0.005	0.00195	0.00094
0.01	0.00281	0.00362
0.015	0.00353	0.00478
0.02	0.00265	0.00372
0.025	0.00508	0.00650
0.03	0.00630	0.00989
0.04	0.00694	0.01101
0.05	0.00992	0.01067
0.1	0.02272	0.01846

Table S6. Contamination prediction error (Mean Absolute Error) on the mixed ethnicity DNA test dataset

Contamination	NgsTroubleFinder Mean Absolute Error (Ethnicity)	VerifyBamID2 Mean Absolute Error (Ethnicity)
0.0	0.00347	0.00020
0.005	0.00345	0.00156
0.01	0.00386	0.00202
0.15	0.00357	0.00216
0.02	0.00455	0.00220
0.025	0.00455	0.00261
0.03	0.00597	0.00327
0.04	0.00651	0.00326
0.05	0.00728	0.00494
0.1	0.01262	0.01175

Table S7. Contamination classification accuracy at varying contamination bin identifying as contaminated samples with a score higher than 0.01 in the RNA dataset.

Contamination Bin	Accuracy	Precision	Recall	VerifyBamID Accuracy	VerifyBamID Precision	VerifyBamID Recall
0.005	0.71795	1.00000	0.26667	0.71795	1.00000	0.26667
0.01	0.84615	1.00000	0.60000	0.84615	1.00000	0.60000
0.015	1.00000	1.00000	1.00000	0.97436	1.00000	0.93333
0.02	1.00000	1.00000	1.00000	1.00000	1.00000	1.00000

Table S8. Contamination classification accuracy at varying contamination bin identifying as contaminated samples with a score higher than 0.01 in the DNA dataset.

Contamination Bin	Accuracy	Precision	Recall	VerifyBamID Accuracy	VerifyBamID Precision	VerifyBamID Recall
0.005	0.51613	0.00000	0.00000	0.51613	0.00000	0.00000
0.01	0.87097	1.00000	0.73333	0.70968	1.00000	0.40000
0.015	1.00000	1.00000	1.00000	0.87097	1.00000	0.73333
0.02	1.00000	1.00000	1.00000	1.00000	1.00000	1.00000

Table S9. Contamination classification accuracy at varying contamination bin identifying as contaminated samples with a score higher than 0.01 in the ethnicity DNA dataset.

Contamination Bin	Accuracy	Precision	Recall	VerifyBamID Accuracy	VerifyBamID Precision	VerifyBamID Recall
0.005	0.65000	1.00000	0.06667	0.62500	0.00000	0.00000
0.01	0.75556	1.00000	0.45000	0.62222	1.00000	0.15000
0.015	0.94000	1.00000	0.88000	0.98000	1.00000	0.96000
0.02	0.96000	1.00000	0.92000	1.00000	1.00000	1.00000
